# Using Gamma and Quantile Regressions to Explore the Association between Job Strain and Adiposity in the ELSA-Brasil Study: Does Gender Matter?

**DOI:** 10.3390/ijerph14111404

**Published:** 2017-11-17

**Authors:** Maria de Jesus Mendes da Fonseca, Leidjaira Lopes Juvanhol, Lúcia Rotenberg, Aline Araújo Nobre, Rosane Härter Griep, Márcia Guimarães de Mello Alves, Letícia de Oliveira Cardoso, Luana Giatti, Maria Angélica Nunes, Estela M. L. Aquino, Dóra Chor

**Affiliations:** 1 Department of Epidemiology and Quantitative Methods in Health, National School of Public Health, Oswaldo Cruz Foundation, Rio de Janeiro 21041-210, Brazil; leticiadeoliveiracardoso@gmail.com (L.d.O.C.); dorachor@gmail.com (D.C.); 2 Department of Nutrition and Health, Federal University of Viçosa, Viçosa 36.570-000, Brazil; leidjaira_lopes@hotmail.com; 3 Laboratory of Health and Environment Education, Oswaldo Cruz Fundation, Rio de Janeiro 21040-900, Brazil; lucia.rotenberg@gmail.com (L.R.); rohgriep@gmail.com (R.H.G.); 4 Scientific Computing Program, Oswaldo Cruz Foundation, Rio de Janeiro 21040-900, Brazil; aline.nobre@fiocruz.br; 5 Institute of Collective Health, Fluminense Federal University, Niterói 24033-900, Brazil; marciagma@id.uff.br; 6 Faculty of Medicine, Federal University of Minas Gerais, Belo Horizonte 30310-100, Brazil; luana.giatti@gmail.com; 7 Pos graduate program in Epidemiology, Federal University of Rio Grande do Sul, Porto Alegre 90035-003, Brazil; maanunes@gmail.com; 8 Institute of Collective Health, Federal University of Bahia, Salvador 40110-040, Brazil; estela@ufba.br

**Keywords:** quantile regression models, adiposity, job strain, body mass index, waist circumference

## Abstract

This paper explores the association between job strain and adiposity, using two statistical analysis approaches and considering the role of gender. The research evaluated 11,960 active baseline participants (2008–2010) in the ELSA-Brasil study. Job strain was evaluated through a demand–control questionnaire, while body mass index (BMI) and waist circumference (WC) were evaluated in continuous form. The associations were estimated using gamma regression models with an identity link function. Quantile regression models were also estimated from the final set of co-variables established by gamma regression. The relationship that was found varied by analytical approach and gender. Among the women, no association was observed between job strain and adiposity in the fitted gamma models. In the quantile models, a pattern of increasing effects of high strain was observed at higher BMI and WC distribution quantiles. Among the men, high strain was associated with adiposity in the gamma regression models. However, when quantile regression was used, that association was found not to be homogeneous across outcome distributions. In addition, in the quantile models an association was observed between active jobs and BMI. Our results point to an association between job strain and adiposity, which follows a heterogeneous pattern. Modelling strategies can produce different results and should, accordingly, be used to complement one another.

## 1. Introduction

Obesity has been associated with occupational factors complementing individual behavioral characteristics relating to calorie intake and physical activity [1]. Job strain is a salient feature of the psychosocial environment, as shown by the classic demand–control model (high psychological demand associated with low job control) [2]. A number of studies have pointed to its association with body mass index (BMI) [3,4,5] and waist circumference (WC) [4,6]. Other studies, however, have found no association with either BMI [7,8,9] or WC [3,10].

This article addresses some analytical considerations raised by studies in this area, which have led to inconsistencies in the results. One of these is how adiposity is measured, because BMI and WC measurements are traditionally considered in categorical forms. Although internationally-agreed cut-off values do exist, these measures are produced on a continuous scale; the loss of information stemming from categorization of the variable can produce inconsistent estimates [11].

While there is an advantage to analyzing outcomes in a continuous form, studies in which the response variable is continuous, asymmetrical and strictly positive (as in the cases of BMI and WC) commonly use the gamma model [12]. The identity link function makes the parameters of the model easier to interpret and the coefficient estimates furnish the mean effect directly. In some situations, however, the effect of the co-variables may not be given by the mean value of the outcome of interest, in which case the gamma regression model may no longer be appropriate. Indeed, a number of studies indicate differences between obese and non-obese individuals as regards response to job strain [1]. For example, when Kivimäki et al. [8] examined BMI in relation to job strain, they observed a tendency to weight gain among the initially overweight, while those with low BMI tended to lose weight.

In the context of that debate, the quantile regression model is advantageous because it allows the co-variables’ effects to be estimated in different quantiles of the distribution; for example, enabling significant effects to be identified in more extreme distribution quantiles [13]. The quantile regression model assumes only that the response variable is continuous. Thus, it combines two advantages: it does not assume a probability distribution for the response variable (as would generally be the case with linear models) and it avoids the loss of information that results from categorizing the dependent variable [14]. Recent studies have used quantile regression models to examine risk factors for adiposity [15,16,17,18], while others have used gamma regression [19,20].

It is also possible for these results to differ by gender, because of both physical and behavioral characteristics. Thus, men with higher BMI subject to highly demanding jobs show greater likelihood of weight gain, while leaner men are more likely to lose weight. The same pattern was not found in women, however [8]. 

No studies evaluating the association between job strain and obesity using quantile regression, and few using gamma regression, have been found to date. This study, therefore, seeks to answer the following questions: Is job strain associated with adiposity when related indicators are examined in continuous form? Is this association heterogeneous as regards the distribution of the outcomes examined? Are the results modified by gender?

This study examines psychosocial job strain in a cohort of workers from the ELSA-Brasil study. The aim was to evaluate the association between job strain and adiposity measured by BMI and WC, using two statistical analysis approaches (gamma regression and quantile regression) and considering the role of gender in the associations.

## 2. Methods

### 2.1. Study Population

The study population was drawn from baseline participants in ELSA-Brasil (2008–2010), a multicenter cohort study accompanying 15,105 active and retired civil servants of both sexes, aged from 35 to 74 years, from teaching and research institutions in six of Brazil’s state capitals: Belo Horizonte, Porto Alegre, Rio de Janeiro, Salvador, São Paulo, and Vitória [21]. Of the 12,096 active participants eligible for this study, the analytical sample included 11,960 (98.9%) for whom complete information was available for the variables analyzed.

### 2.2. Job Strain

The exposure variable was measured using the Brazilian version of the Swedish Demand–Control–Support questionnaire [22] based on the Job Content Questionnaire [23]. The Demand–Control–Support questionnaire spans three dimensions: psychological demands (five items), control (six items), and social support at work (six items), and displayed acceptable psychometric properties in the Brazilian context [24,25]. It was considered reliable and highly correlated with the Job Content Questionnaire [23].

Job strain was evaluated using the quadrants proposed by Karasek (1979) [2]. In order to form the quadrants, the scores obtained in the psychological demands and control dimensions were categorized by their median in the study population by sex (14 and 13 points for psychological demands among women and men, respectively, and 18 points for control in both sexes). The quadrants were: low strain (low demand/high control; reference category), passive job (low demand/low control), active job (high demand/high control), and high strain (high demand/low control).

### 2.3. BMI and WC

BMI was calculated as weight (kg) divided by height squared (m^2^). Body weight (kg) was measured on electronic scales (Toledo), with maximum capacity of 200 kg and precise to 0.1 kg; height (cm) was measured with a fixed stadiometer (SECA-SE-216) and a scale to 0.1 cm. WC (cm) was measured using a 150-cm millimetric tape (*Gulick* model, *Mabis*) or 200-cm steel measuring tape, applied directly on the skin and taken at the mid-point between the lower border of the costal margin (excluding the floating ribs) and the iliac crest on the mid-axillary line. All measurements were taken according to established techniques [26]. These two variables were examined in continuous form.

### 2.4. Co-Variables

The co-variables examined were: age (continuous); schooling (up to fundamental education, elementary school, higher education and postgraduate studies); marital status (married/cohabiting, divorced/separated/widowed and single); hours worked weekly (continuous); study center (Belo Horizonte, Porto Alegre, Rio de Janeiro, Salvador, São Paulo and Vitória); and standardized per capita family income (continuous). As there were major variations in this latter variable, each value was standardized by subtracting from the mean and dividing by the standard deviation (SD).

### 2.5. Statistical Analysis

For the descriptive analyses, categorical variables were expressed as percentages and continuous variables, as means, SD, and quartiles. The associations between the study variables were estimated using gamma regression models with an identity link function, because the outcomes are continuous and asymmetrical [12]. Firstly, these models were estimated to investigate the crude association of job strain and the co-variables with BMI and WC. Then, the adjusted associations of job strain with the two outcomes evaluated were estimated by testing the introduction of each of the potential confounders from the crude model. The criteria used to assess the importance of each co-variable in the model were the Akaike Information Criterion (AIC) [27] and the deviance statistic (chi-square test), the least values of which were considered to indicate the best fit. From the final set of co-variables established by gamma regression, quantile regression models were also estimated. These models are appropriate when the response variable distribution is asymmetrical and allow the effect of the independent variables to be observed in different quantiles of the response variable distribution [13]. Fixed intervals of five quantiles, starting at the 5th and up to the 95th quantile, were used to estimate the coefficients.

On the basis of a hypothesis established a priori, which was corroborated by significant tests of interaction (*p* < 0.05), all the analyses were conducted separately by sex. In all the analyses, the level of confidence was set at 95% and the statistical software used was R version 3.1.2 [28]. The *quantreg* library was deployed to fit the quantile regression models and *ggplot2* was used for the graphs.

### 2.6. Ethical Considerations

The ELSA-Brasil study was approved by the National Research Ethics Commission (CONEP; No. 976/2006) and by the research ethics committees of each of the participating institutions. All participants signed a declaration of informed consent.

## 3. Results

### 3.1. Descriptive Characteristics

Participant mean age was similar among men and women. The women displayed higher levels of schooling, higher per capita family income, were married or, less often, cohabiting. As regards their occupational characteristics, the women were more often classified as in passive jobs and reported levels of social support at work similar to those of the men. A longer working week was observed among the men (Table 1). 

The women’s mean BMI was 26.9 kg/m^2^ (1st quartile [Q1] = 23.2; median [Q2] = 26; 3rd quartile [Q3] = 29.8), and the men’s was 27 kg/m^2^ (Q1 = 24.1; Q2 = 26.5; Q3 = 29.4). The WC values for men and women were 87 cm (Q1 = 78; Q2 = 85.3; Q3 = 94.4) and 94.9 cm (Q1 = 87; Q2 = 94.2; Q3 = 102), respectively (data not shown in Table 1 and Table 2).

### 3.2. Unadjusted Association of Variables Examined with BMI and WC

In both sexes, age and being divorced, separated, or widowed, rather than single, displayed positive associations with BMI and WC. That same positive association was observed, among men only, for those married or cohabiting. Higher levels of schooling and income were inversely associated with BMI and WC among women. For example, as compared with women with less than complete secondary schooling, women with postgraduate qualifications showed 2.97 kg/m^2^ lower BMI and 6.93 cm smaller WC. Among the men, meanwhile, having postgraduate qualifications was associated with 1 cm larger WC. Among the women, hours worked weekly and WC showed an inverse association, while among the men, the association was direct. As compared with low strain, a passive job was associated with 0.77 kg/m^2^ greater BMI and 1.90 cm larger WC among the women, but 1.04 cm smaller WC among the men. A high strain was positively associated with BMI in both men and women, but directly associated with WC among women only (Table 1).

### 3.3. Adjusted Association of Job Strain with BMI and WC—Results for Women

In the gamma models (Table 2), passive and high-strain jobs continued to be significantly associated with the two outcomes after adjustment for age. After adjustment for schooling, however, no association was found between psychosocial job strain and BMI or WC.

**Figure 1:**
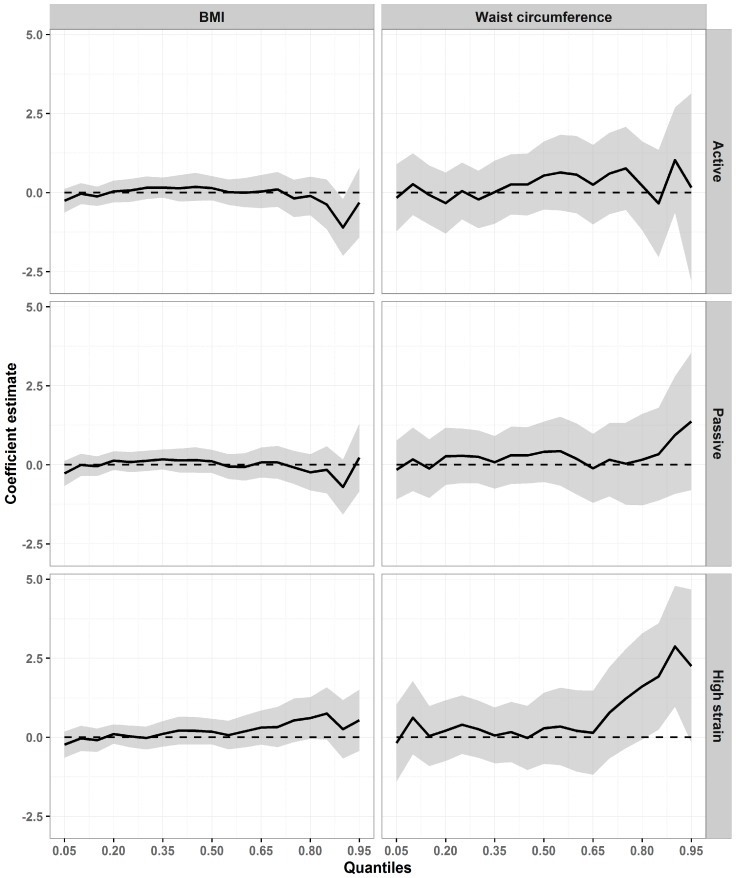
Association between job strain and adiposity (BMI and WC) in adjusted quantile regression models among women. ELSA-Brasil, 2008–2010 (*n* = 6252). On the horizontal axes are the BMI and WC distribution percentiles; the vertical axes show the values of the coefficients estimated. The dashed parallel line represents the null value (zero), and a solid line stands for quantile estimates. The grey area surrounding the solid line represents the 95% CI for the quantile estimates. The coefficients are adjusted by age, schooling, per capita family income, hours worked weekly, and study center for BMI; and by age, schooling, per capita family income, and study center for WC.

In the quantile models for women, differing patterns of association were identified across the distributions of the two outcomes. Figure 1 shows a tendency for the effects of high job strain to increase in the upper quantiles of both distributions (from quantile 60 for BMI and quantile 65 for WC). For example, for WC, the highest coefficient was seen in quantile 90 (2.87 cm; 95% confidence interval [CI]: 0.96, 4.79), an increase of nearly 1 cm in relation to the coefficient of the previous quantile (1.92 cm; 95% CI: 0.24, 3.61). That means that, taking low strain as the reference, high job strain was associated with a 1.92 cm and 2.87 cm increase in WC in quantiles 85 and 90 of the distribution, respectively.

### 3.4. Adjusted Association of Job Strain with BMI and WC—Results for Men

In the gamma models (Table 2), BMI was observed to be 0.54 kg/m^2^ (95% CI: 0.21, 0.87) higher in men classified as under high job strain, as compared with those under low strain, after adjustment for the co-variables. WC was 1.12 cm (95% CI: 0.18, 2.06) greater in the high-strain category than in the low-strain category.

**Figure 2:**
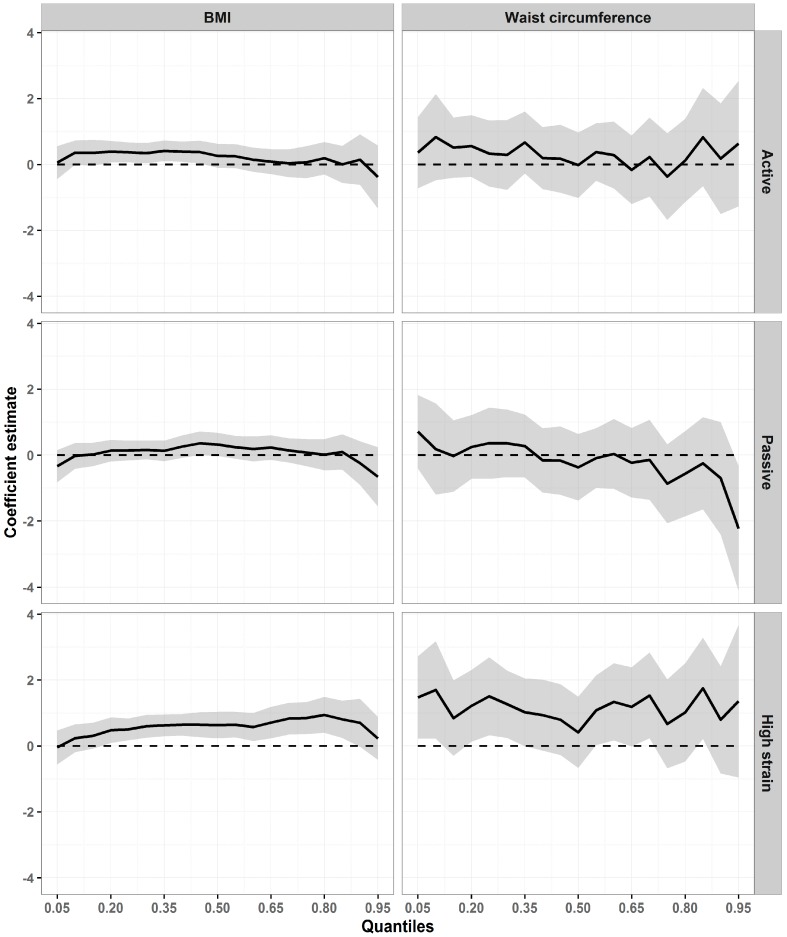
Association between job strain and adiposity (BMI and WC) in adjusted quantile regression models among men. ELSA-Brasil, 2008–2010 (*n* = 5708). On the horizontal axes are the BMI and WC distribution percentiles; the vertical axes show the values of the coefficients estimated. The dashed parallel line represents the null value (zero), and a solid line stands for quantile estimates. The grey area surrounding the solid line represents the 95% CI for the quantile estimates. The coefficients are adjusted by age, marital status, and study center for BMI; and by age, marital status, schooling, hours worked weekly, and study center for WC.

In the quantile models (Figure 2), a heterogeneous pattern of association was also observed across the distributions of each of the two outcomes. In comparison with the low-strain quadrant, an active job was positively associated with BMI: the coefficients were 0.39 kg/m^2^ (95% CI: 0.06, 0.72) in quantile 20, 0.42 kg/m^2^ (95% CI: 0.11, 0.72) in quantile 35, and 0.38 (95% CI: 0.03, 0.72) in quantile 45. A tendency was also observed for the effects of high job strain on BMI to increase: coefficients ranged from 0.48 kg/m^2^ (95% CI 0.09, 0.87) in quantile 20 to 0.94 kg/m^2^ (95% CI: 0.4, 1.49) in quantile 80. WC also displayed a heterogeneous pattern of association with high job strain across the distribution: the highest coefficient was observed in quantile 85 (1.75 cm; 95% CI: 0.22, 3.29).

## 4. Discussion

This study examined adiposity by way of BMI and WC, on a continuous scale, using gamma regression and quantile regression models, two approaches as yet little applied to epidemiology. In the fitted gamma models, among the women, no association was observed between job strain and adiposity. In the quantile models, however, the pattern observed was for the effects of high job strain to increase in the upper quantiles of the BMI and WC distributions. Among the men, high strain was associated with adiposity in the gamma regression models. However, when quantile regression was used, the association was observed not to be homogeneous across the distributions of the two outcomes. In addition, an association between an active job and BMI was observed only in the quantile models. These results indicate that the strategies used were appropriate, because the associations detected might not have been perceived had we used only traditional regression models that estimate mean effects.

Although the comparison between these results and those of other studies is restricted by the differences in the methods used, the fact is that the association between job strain and adiposity is still controversial. Some prospective studies have not found any association between job strain and adiposity [8,29]. A systematic review by Kivimäki and Kawachi (2015) showed that the results are inconsistent. Nyberg et al. (2012) examined 160,000 adult participants in 13 cohort studies, finding a statistically significant association between higher job strain and higher BMI. Some cross-sectional studies, however, have reported an association between job strain and higher BMI among women [30,31]. In a study of Japanese employees, Ishizaki et al. (2008) found no statistically significant association between job strain and BMI or waist-to-hip ratio.

Job strain can foster increased adiposity directly by chronic activation of the hypothalamic–pituitary–adrenal axis, thus raising cortisol secretion levels [32]. This hormone can induce weight gain by heightening appetite, as well as by mobilizing peripheral fat towards the abdominal region [33,34]. Job strain can also act indirectly to favor the adoption of inappropriate health-related behavior associated with weight gain [35]. Excessive demands combined with a lack of control over the work process [36] and long working weeks [37] have been associated with lower levels of leisure-time physical activity. Additionally, individuals chronically exposed to stress tend to engage in food-related risky behavior characterized by over-eating [38] and by ingestion of high energy-density foods [39], which are regarded as ‘comfort foods’, because they attenuate physiological responses to stress [40]. Lastly, job-environment stressors can favor weight gain through excessive ingestion of alcohol [41] and inappropriate sleep patterns [42].

Our results indicated more consistent associations between job strain and adiposity among men than among women, as has been observed in other studies [43,44]. Although the mechanisms that explain this difference in patterns between men and women are still not clear, it has been proposed that gender differences may attenuate or heighten the effects of job strain on adiposity [45]. In addition to differences in working conditions [46], women are more involved in caring for home and children [47] and experience greater work–family conflict [48]. This accumulation of family and social roles may make it more complex to study the relationship between job strain and adiposity in women.

It was also observed here that the effects of high job strain are greater in the upper quantiles of the distributions. This characteristic pattern of association has been observed previously with other determinants of adiposity, such as sedentary behavior [49], eating out [15], genetic factors [50], and occupational factors, such as night work and domestic overwork [18]. On the basis of this evidence, it has been suggested that obesity itself may constitute one important component of the obesogenic environment, acting to increase sensitivity to various risk factors associated with weight gain [51].

It is important to note that the inconsistencies in our findings and in the literature may be due, in part, to the many different ways job strain is investigated, from the form in which it is analyzed through to how it is operationalized [52,53]. Another point is the heterogeneity in outcome, because the association is often present in specific portions of the distribution of the outcome analyzed and, when that association is evaluated overall (as in gamma regression), it tends to disappear. The two approaches used in this study examine the outcome in continuous form, eliminating possible problems of categorization [14]. Different possible modelling options exist when considering a continuous outcome. Most used is the linear regression model, but its assumption of normality is not always appropriate, particularly when considering an asymmetrical distribution. In that case, the regression model with gamma distribution may be more suitable. However, generalized linear models (as is the case with gamma distribution) estimate the mean effect of the explanatory variables on the outcome [12]. If there is heterogeneity in the outcome, the quantile regression model becomes more appropriate, because it allows the effects to vary by different quantiles in the distribution [13]. When the distribution is asymmetrical, this advantage becomes even more evident, because the mean may not be the most suitable measure for distributions of this kind.

One limitation of this study is the cross-sectional nature of the analyses, because the temporality of the associations studied cannot be assured. However, there is little likelihood that the degree of adiposity modifies the degree of exposure to job strain.

The main strength of this study is its originality in the analytical methods used and also the use of two different measures of adiposity. Another important point is the quality control in gathering the anthropometric values measured for the study. Before beginning field work, the team of measurement-takers was centrally trained and certified. During data collection, local team meetings were held to discuss problems and for systematic supervision and recertification [54].

## 5. Conclusions

The results of this study, even after adjustment for important socioeconomic variables, such as income and schooling, point to an association between job strain and adiposity evaluated by way of BMI and WC, which displayed a heterogeneous pattern, that is, varying in different portions of the outcome distributions by sex. As observed in this study, modelling strategies can produce different results and, accordingly, should be used complementarily, particularly when variables such as BMI and WC are involved.

## Figures and Tables

**Table 1:** Sample characteristics and crude associations of body mass index (BMI) and waist circumference (WC) with variables examined. ELSA-Brasil, 2008–2010 (*n* = 12,096).

Variables	Women (*n* = 6316)			Men (*n* = 5780)		
	Sample Characteristics *n* (%) or Mean (SD)	Coefficient (95% Confidence Interval, CI) ^a^		Sample Characteristics *n* (%) or Mean (SD)	Coefficient (95% CI) ^a^	
		BMI	Waist Circumference		BMI	Waist Circumference
**Age**	48.9 (7.1)	0.07 (0.06, 0.09)	0.31 (0.27, 0.35)	49.6 (7.5)	0.02 (0.00, 0.03)	0.20 (0.16, 0.24)
**Schooling**						
<Secondary complete	462 (7.3)	Reference	Reference	871 (15.1)	Reference	Reference
Secondary complete	2345 (37.1)	−1.13 (–1.67, −0.61)	−3.61 (–4.90, −2.33)	2043 (35.3)	−0.18 (–0.52, 0.16)	−0.36 (–1.28, 0.56)
Undergraduate complete	1223 (19.4)	−2.31 (–2.87, −1.76)	−6.28 (–7.65, −4.93)	714 (12.4)	−0.27 (–0.69, 0.16)	0.06 (–1.09, 1.22)
Postgraduate	2286 (36.2)	−2.97 (–3.50, −2.45)	−6.93 (–8.21, −5.66)	2152 (37.2)	−0.34 (–0.68, 0.00)	1.00 (0.08, 1.92)
**Per capita family income**	837.8 (675.5)	−0.60 (–0.71, −0.49) ^b^	−1.23 (–1.51, −0.94) ^b^	786.50 (639.9)	−0.11(–0.22, 0.01) ^b^	0.27 (–0.04, 0.59) ^b^
**Marital status**						
Single	893 (14.1)	Reference	Reference	323 (5.6)	Reference	Reference
Divorced/separated/widowers	1945 (30.8)	0.50 (0.10, 0.90)	2.12 (1.14, 3.10)	774 (13.4)	0.59 (0.04, 1.14)	2.08 (0.59, 3.55)
Married/living together	3477 (55.1)	0.06 (–0.31, 0.43)	0.54 (–0.36, 1.44)	4683 (81.0)	0.86 (0.39, 1.33)	3.07 (1.78, 4.34)
**Hours worked weekly**	42.0 (10.1)	0.00 (–0.02, 0.01)	−0.04 (–0.07, −0.01)	44.6 (11.3)	0.01 (0.00, 0.02)	0.04 (0.02, 0.07)
**Quadrants**						
Low strain	1468 (23.3)	Reference	Reference	1393 (24.2)	Reference	Reference
Active	1213 (19.3)	−0.07 (–0.44, 0.31)	0.02 (–0.91, 0.95)	1424 (24.7)	0.11 (–0.21, 0.42)	0.20 (–0.67, 1.07)
Passive	2280 (36.3)	0.77 (0.45, 1.10)	1.90 (1.09, 2.71)	1714 (29.8)	−0.03 (–0.33, 0.28)	−1.04 (–1.86, −0.21)
High strain	1328 (21.1)	0.78 (0.41, 1.16)	1.49 (0.58, 2.41)	1227 (21.3)	0.44 (0.11, 0.77)	0.39 (–0.51, 1.30)
**Social support at work**	19.5 (3.3)	0.02 (–0.02, 0.06)	0.08 (–0.02, 0.17)	20.0 (3.3)	−0.02 (–0.05, 0.02)	−0.04 (–0.13, 0.05)

^a^ Unadjusted analysis using gamma regression. ^b^ Coefficients for standardized per capita family income.

**Table 2:** Coefficients and respective 95% confidence interval (CI) of the association between job strain and adiposity (BMI and WC) in adjusted gamma regression models by gender. ELSA-Brasil, 2008–2010 (*n* = 11,960).

Adjusted Models	Coefficient (95% CI)			Difference in Deviance
	Quadrants			
	Active	Passive	High Strain	
**Women (*n* = 6252)**				
**BMI**				
Model 1: age	−0.11 (−0.48, 0.27)	0.74 (0.41, 1.07)	0.88 (0.50, 1.25)	2.585
Model 2: model 1 + schooling	0.01 (−0.36, 0.38)	0.01 (−0.33, 0.35)	0.26 (−0.12, 0.64)	5.818
Model 3: model 2 + per capita family income	0.03 (−0.34, 0.40)	−0.06 (−0.40, 0.29)	0.19 (−0.19, 0.57)	0.679
Model 4: model 3 + hours worked weekly	−0.09 (−0.47, 0.28)	−0.04 (−0.39, 0.30)	0.15 (−0.24, 0.53)	0.442
Model 5: model 4 + study center	−0.07 (−0.45, 0.30)	0.02 (−0.33, 0.36)	0.23 (−0.15, 0.61)	1.014
**Waist circumference**				
Model 1: age	−0.10 (−1.02, 0.81)	1.80 (1.00, 2.60)	1.88 (0.97, 2.78)	4.171
Model 2: model 1 + schooling	0.08 (−0.83, 0.99)	0.47 (−0.36, 1.31)	0.75 (−0.17, 1.68)	1.943
Model 3: model 2 + per capita family income	0.12 (−0.79, 1.03)	0.29 (−0.55, 1.13)	0.56 (−0.37, 1.49)	0.485
Model 4: model 3 + study center	0.21 (−0.69, 1.12)	0.29 (−0.55, 1.12)	0.76 (−0.17, 1.69)	1.333
**Men (*n* = 5708)**				
**BMI**				
Model 1: age	0.13 (−0.19, 0.45)	0.01 (−0.30, 0.31)	0.51 (0.18, 0.84)	0.112
Model 2: model 1 + marital status	0.13 (−0.19, 0.45)	0.02 (−0.28, 0.32)	0.51 (0.17, 0.84)	0.344
Model 3: model 2 + study center	0.15 (−0.17, 0.46)	0.03 (−0.27, 0.33)	0.54 (0.21, 0.87)	1.541
**Waist circumference**				
Model 1: age	0.38 (−0.49, 1.24)	−0.86 (−1.68, −0.03)	0.74 (−0.15, 1.64)	1.351
Model 2: model 1 + marital status	0.38 (−0.49, 1.24)	−0.82 (−1.64, 0.00)	0.75 (−0.15, 1.65)	0.262
Model 3: model 2 + schooling	0.29 (−0.58, 1.16)	−0.29 (−1.17, 0.58)	1.21 (0.27, 2.15)	0.182
Model 4: model 3 + hours worked weekly	0.15 (−0.73, 1.03)	−0.27 (−1.15, 0.60)	1.13 (0.19, 2.07)	0.061
Model 5: model 4 + study center	0.19 (−0.68, 1.06)	−0.30 (−1.17, 0.57)	1.12 (0.18, 2.06)	1.095
